# Enhancing pre-trained language model by answering natural questions for event extraction

**DOI:** 10.3389/frai.2025.1520290

**Published:** 2025-04-24

**Authors:** Yuxin Zhang, Qing Han

**Affiliations:** School of Medical Technology and Information Engineering, Zhejiang Chinese Medical University, Hangzhou, China

**Keywords:** event extraction, pre-trained language model, topic words, question answering, contextual information

## Abstract

**Introduction:**

Event extraction is the task of identifying and extracting structured information about events from unstructured text. However, event extraction remains challenging due to the complexity and diversity of event expressions, as well as the ambiguity and context dependency of language.

**Methods:**

In this paper, we propose a new method to improve the precision and recall of event extraction by including topic words related to events and their contexts, directing the model to focus on the relevant information, and filtering the noise.

**Results:**

This method was evaluated on the ACE 2005 dataset, achieving an F1-score of 77.27% with significant improvements in both precision and recall.

**Discussion:**

Our results show that the use of topic words and question answering techniques can effectively address the challenges faced by event extraction and pave the way for the development of more accurate and robust event extraction systems.

## 1 Introduction

Events are things that happen or occur, and usually involve entities (people, time, place, etc.) as their properties. Understanding events based on their descriptions in text is essential for machine reading systems. It is also useful in many practical applications such as news summarization, information retrieval, question answering, knowledge base construction, etc. (Li et al., 2019). As one of the main tasks in information extraction area, the aim of the Event Extraction (EE) task is to extract structured event information from unstructured text (Hogenboom et al., 2016). These tasks were identified in general as the extraction of the entities, relations and events being discussed in the language (Doddington et al., 2004). Event Extraction (EE) is a complex yet crucial Natural Language Processing (NLP) technology. This technology not only focuses on identifying the entities mentioned in the text (such as names of people, places, organizations, etc.), but also delves deeper into the specific events that occur between these entities and the attributes of these events, such as time, location, participants, and so on.

Following the event extraction task definition in ACE 2005, an event is frequently described as a change of state, indicating a specific occurrence of something that happens in a particular time and a specific place involving one or more participants. It can help answer the “5W1H” questions, i.e., “who,” “when,” “where,” “what,” “why” and “how” about an event Liu et al. (2021) proposed a new event extraction paradigm, which transforms the event extraction task into a question answering (QA) task, extracts event arguments in an end-to-end manner, significantly improves the error propagation problem in traditional methods, and demonstrates its ability in zero-shot learning settings. However, this method heavily relies on pre-trained language models such as Bidirectional Encoder Representations from Transformers (BERT) to obtain contextual representations of input sequences.

Large language models, including BERT, GPT, and their derivatives, have fundamentally transformed the landscape of NLP by show-casing exceptional abilities in comprehending and generating human language (Han and Zhao, 2025). These models, nourished by vast quantities of textual data, are adept at capturing intricate linguistic patterns and relationships. Consequently, they have emerged as indispensable assets for a myriad of NLP tasks, particularly event extraction. Large language models offer richer, more contextualized representations of words and phrases, which are pivotal for precisely pinpointing and comprehending entities and the events they engage in. They possess the capability to discern subtle nuances in language that might escape the notice of traditional methodologies. Pre-trained large language models can be tailored for specific tasks, such as event extraction, by leveraging the knowledge amassed during their pre-training phase. This transfer learning methodology often expedites the training process and enhances performance compared to starting from scratch. Moreover, these models excel at navigating complex event structures and relationships. They comprehend the intricate interplay among multiple entities, actions, and attributes within an event, facilitating more accurate extraction of event-related information. Scalable in nature, these models can handle larger volumes of data and generalize to new domains or event types with minimal additional training, making them particularly suited for event extraction tasks that encompass diverse and ever-evolving event types. However, despite their prowess in numerous NLP tasks, the performance of pre-trained models is still bound by the quality of their training data and model capacity. Hence, for the extraction of events within niche domains or rare occurrences, this methodology might not yield optimal outcomes.

We acknowledge that the limitations of existing research in the realm of event extraction primarily stem from an insufficient utilization of contextual information. Contextual cues play a pivotal role in understanding the nuances and intricacies of language, yet they are often overlooked or underrepresented in current methodologies (Zeng et al., 2019). This oversight has direct consequences on the accuracy rates achieved by event extraction systems, as they struggle to fully grasp the semantic and pragmatic meanings embedded within textual data.

Our study is motivated by the urgent need to address this gap by enhancing the contextual awareness of event extraction models. Topic words are the most representative or high-probability words associated with a latent topic discovered from documents (Han, 2024). We hypothesize that by incorporating topic words—key terms or phrases that summarize the broader context surrounding an event—we can significantly boost the model’s ability to comprehend and interpret event-related information. These topic words serve as bridges, connecting individual events to their larger narrative contexts, enabling the model to capture a more holistic view of the events it encounters.

The inclusion of topic words is expected to bring several benefits:

Improved accuracy: by leveraging contextual information embodied in topic words, the model can make more informed decisions about event boundaries, types, and arguments, resulting in a noticeable improvement in extraction accuracy.

Robustness: the model’s ability to generalize to new domains or event types will be enhanced, as topic words can provide a high-level understanding of the context that transcends specific instances or examples.

Scalability: as the model becomes more adept at capturing contextual nuances, it can effectively handle larger volumes of data with diverse event types, making it more scalable for real-world applications.

Interpretability: the use of topic words can also improve the interpretability of the model’s predictions, as they offer a transparent window into the contextual factors that influenced the extraction process.

In summary, the motivation behind this study is to address the inadequacy of contextual information in existing event extraction research and to propose a novel approach that incorporates topic words to summarize and leverage this crucial information. By doing so, we aim to advance the field of event extraction and enable the development of more accurate, robust, scalable, and interpretable models.

The contribution of this study is multifaceted and significantly advances the landscape of event extraction research. By introducing a novel paradigm that integrates topic words into the input sequence, we have made several noteworthy contributions to addressing the challenge of contextual information insufficiency:

Enhanced contextual awareness: at the core of our approach lies a strategic innovation that enriches the model’s understanding of the underlying context. By seamlessly incorporating topic words into the input sequence, we provide the model with a broader and deeper perspective of the textual data, enabling it to grasp nuanced meanings and relationships that might otherwise be overlooked. This additional contextual information acts as a catalyst, amplifying the model’s extraction performance and yielding more accurate and comprehensive results.

Label-efficient learning: in a departure from conventional wisdom that often relies heavily on labeled data, our model demonstrates remarkable resilience and effectiveness without the need for additional labeled data specifically for extraction. This label-efficient feature is particularly advantageous given the scarcity and high cost of acquiring large-scale, high-quality labeled datasets. By leveraging the inherent information within the text itself, our approach circumvents this barrier and paves the way for more practical and scalable solutions.

In this paper, we present a comprehensive survey of enhancing event extraction through incorporating topic words.

The first section introduces the problem of event extraction and its challenges. It outlines the importance of event extraction in various applications such as news summarization, information retrieval, and knowledge base construction. Furthermore, it highlights the complexity and diversity of event expressions, as well as the ambiguity and context-dependency of language, which pose significant obstacles to effective event extraction.

Next, we delve into the main knowledge and techniques that underpin our proposed approach. We explain how we formulate event extraction as a question answering task, leveraging the power of pre-trained language models like BERT. By incorporating topic words related to the event and its context, we aim to guide the model to focus on relevant information and filter out noise, thereby improving the precision and recall of event extraction.

In section 2, we provide a detailed methodology outlining the key steps and components of our approach. This includes preprocessing techniques to ensure the model understands the basic units of text, the extraction of topic words to summarize contextual information, and the construction of informative input sequences tailored to specific event types and their arguments. We also discuss the use of special tokens and positional encoding to facilitate the model’s understanding of the input sequence.

Section 3 focuses on the experiments conducted to evaluate the performance of our proposed approach. We describe the dataset and evaluation metrics used, and present the results of our experiments in comparison to a benchmark model without topic words. The results demonstrate the significant improvements achieved by incorporating topic words into the input layer of the event extraction model, with notable gains in precision, recall, and F1 scores.

In the last part of the paper, we discuss the related work in the field of event extraction and pre-trained language models. We compare and contrast our approach with existing methods, highlighting the unique advantages and contributions of our study. Finally, we conclude by summarizing the main findings and outlining the potential applications and future directions of our research.

## 2 Related works

### 2.1 Event extraction

Most existing event extraction methods primarily focus on extracting event arguments within the scope of individual sentences (Ji and Grishman, 2008). While these methods have achieved notable success in sentence-level tasks, they often struggle in scenarios where event arguments are distributed across multiple sentences or documents. Specifically, two major challenges arise: (1) event arguments of the same event are frequently scattered across different sentences, leading to incomplete extraction results, and (2) multiple sentences or documents may describe the same event, resulting in redundant or overlapping information. These limitations highlight the need for more advanced techniques capable of handling document-level and corpus-level event extraction, which involve addressing long-term dependencies and resolving entity and event coreference issues (Liu et al., 2021).

Recent advancements in document-level event extraction have sought to address these challenges by leveraging graph-based neural networks and hypergraph structures. For instance, Zhao et al. (2020) proposed a dependency-based Graph Convolutional Network (GCN) to capture local contextual information within sentences, combined with a hypergraph to model global dependencies across documents. This approach was further enhanced by the introduction of stacked Hypergraph Aggregation Neural Network (HANN) layers, which enable fine-grained interactions between local and global contexts (Liu et al., 2021). While these methods have shown promise in capturing complex relationships, they often rely heavily on information redundancy within large corpora, making them less effective when applied to smaller datasets or documents with limited context.

In contrast to these approaches, our work introduces a novel perspective by framing event extraction as a “grounding” task, leveraging the rich semantics of event types and their associated argument roles. Unlike traditional methods that depend on large-scale data redundancy, our approach infers event types and argument roles by clustering similar events, enabling it to operate effectively even with limited input. This makes our method particularly suitable for scenarios where the input consists of only a few sentences or documents. Furthermore, our approach is complementary to existing paradigms, as it grounds each event cluster to a predefined event ontology (Huang et al., 2017), providing a structured and interpretable framework for event extraction.

Recent studies have also explored the integration of external knowledge and pre-trained language models to enhance event extraction. For example, Liu et al. (2020) proposed a hybrid model combining BERT with external knowledge graphs, achieving state-of-the-art performance. Similarly, Zhao et al. (2019) introduced a multi-task learning framework that jointly optimizes entity recognition and event extraction, demonstrating significant improvements in cross-sentence event coreference resolution. While these methods have advanced the field, they often require extensive computational resources and large annotated datasets, limiting their applicability in resource-constrained settings.

Our method addresses these limitations by incorporating topic words as contextual cues, which guide the model to focus on relevant information while filtering out noise. This not only improves the accuracy of event extraction but also enhances the model’s ability to generalize across different domains and event types. By analyzing the topics of event-containing citations, we reduce the variance and sparsity associated with simple keyword searches, ensuring that the extracted events are both precise and contextually relevant. Additionally, our use of processed events as subject terms eliminates the ambiguity often introduced by manual annotation, further reducing category redundancy and improving the overall quality of the extraction results.

In summary, while existing methods have made significant strides in event extraction, they often fall short in handling cross-sentence dependencies and require large datasets to achieve optimal performance. Our approach, by contrast, offers a more flexible and scalable solution, capable of extracting events from corpora of any size while maintaining high precision and recall. By integrating topic words and leveraging the semantic richness of event types, we provide a robust framework that complements existing methods and addresses their key limitations.

### 2.2 Pre-trained language model for event extractions

#### 2.2.1 Pre-trained language model

Pre-trained language models are capable of capturing the meaning of words dynamically in consideration of their context (Yang et al., 2019). In the field of Event Extraction, the application of pre-trained models has greatly improved the accuracy and efficiency of event extraction. These models are mainly based on deep learning methods, especially the Transformer architecture in Natural Language Processing NLP.

#### 2.2.2 BERT and its variants

BERT stands out as one of the most influential pre-trained models in recent NLP research. BERT is conceptually simple, powerful and widely used. It has achieved state-of-the-art results in 11 natural language processing tasks (Devlin et al., 2018). By leveraging large-scale unannotated text for pre-trained, BERT learns rich contextualized representations that can be effectively fine-tuned for various downstream tasks, including Event Extraction. The bidirectional nature of BERT’s attention mechanism allows it to capture contextual information from both directions, which is crucial for identifying event triggers and their arguments accurately.

Several variants of BERT have been proposed to further improve its performance. A Robustly Optimized BERT Pretraining Approach (RoBERTa) introduces optimizations to the pre-trained procedure of BERT, including a larger training dataset, longer training time, and dynamic masking, leading to better performance on Event Extraction and other NLP tasks (Liu et al., 2019). A Lite BERT for Self-supervised Learning of Language Representations (ALBERT) focuses on reducing BERT’s model size while maintaining its performance through techniques such as parameter sharing and embedding factorization, making it a suitable choice for resource-constrained scenarios (Lan et al., 2020). A comprehensive comparative analysis of transformer models, including BERT and its variants, such as RoBerta, Albert, and DistilBert (Sanh et al., 2019), has been conducted to evaluate their performance in addressing long-form question-answering tasks. Among them, BERT and RoBerta proved to be the best performing models for this task, with accuracies of 87.2 and 86.4%, respectively (Nitish et al., 2022).

#### 2.2.3 ELECTRA and other pre-trained models

Efficient Learning Encoder for Accurate Classification of Replaced Tokens (ELECTRA) proposes a new pre-training objective called replaced token detection, which consists of training a generator to replace a small portion of tokens in the input and a discriminator to distinguish replaced tokens from the original tokens. Masked Language Modeling (MLM) pre-training methods in BERT require a large amount of computation (Clark et al., 2020). This approach is more efficient because the task is defined over all tokens, not just the part of the token that is masked out. This approach is also effective in large-scale applications. ELECTRA can be trained much faster than BERT, and is comparable to the performance of RoBERTa and XLNet, but with <1/4 of their computational effort. This approach allows ELECTRA to achieve better performance with fewer pre-training steps compared to BERT. ELECTRA has shown promising results in event extraction, proving its effectiveness in capturing semantic nuances. ELECTRA has achieved promising results in event extraction, proving its effectiveness in capturing semantic nuances.

In addition to BERT and its variants, other pre-trained models have also been explored for Event Extraction. Enhanced Representation through kNowledge Integration (ERNIE) (Sun et al., 2019), developed by Baidu, incorporates external knowledge graphs into the pre-trained process, enhancing the model’s ability to understand complex semantic relationships. This capability is particularly useful for Event Extraction, where events often involve intricate interactions among multiple entities and concepts.

In summary, the advent of pre-trained language models, especially those based on the Transformer architecture, has revolutionized the field of Event Extraction. Models like BERT, RoBERTa, ALBERT, and ELECTRA have demonstrated their prowess in extracting event-related information from unstructured text, pushing the boundaries of what is possible in this domain. As research continues to evolve, we expect to see even more advanced pre-trained models that further enhance the performance of Event Extraction systems.

## 3 Methods

**Figure 1:**
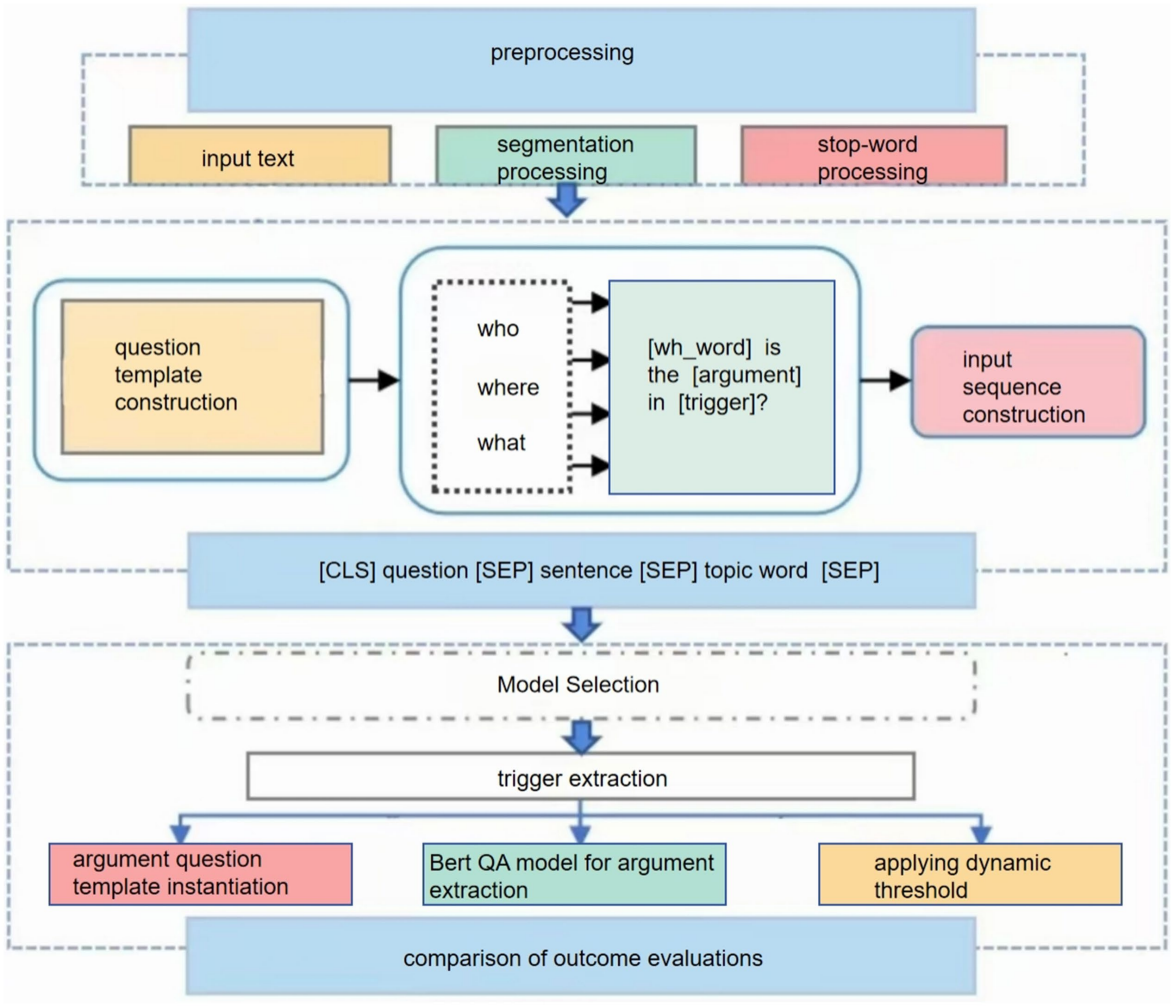
Framework diagram.

In this section, we first provide the overall framework diagram (Figure 1), then discuss each step in the framework in depth.

### 3.1 Overview of the methodology

Preprocessing: segmentation processing ensures that the model understands the basic units of the text, while deactivation and stemming extraction help to reduce lexical diversity and make it easier for the model to capture useful features.

Subject words extraction: accurate extraction of subject words is crucial for subsequent tasks. We choose the event processed by TF-IDF as the subject words to ensure the accuracy and representativeness of the subject words.

Problem template for parameter extraction: we chose a question template based on the annotation guide (Du and Cardie, 2020). The ACE Event Annotation Guidelines provide parameter role descriptions (Linguistic Data Consortium, 2015), using which we can design more natural questions and incorporate more semantic information.

Input sequence construction: the design of placing the topic words at the end of the sequence is based on the following considerations: while the BERT model is able to capture bi-directional contextual information when processing sequences, the beginning and the end of the sequence usually have an impact on the allocation of the model’s attention. Placing subject terms at the end allows the model to focus on this key information after processing the main body of the document, thus potentially better integrating global and local information.

Use of the (SEP) separator: (SEP) is a special token predefined by BERT to separate different paragraphs or sentences in an input sequence. Here, we use it to clearly distinguish between the main content of the input sentence and the topic words, helping the model to better understand the structure of the input sequence.

### 3.2 Choice of topic words

TF-IDF is a statistical method for assessing the importance of a word to a set of documents or to one of the documents in a corpus (Mishra and Vishwakarma, 2015). The importance of a word increases proportionally with the number of times it appears in a document, but decreases inversely with the frequency of its appearance in the corpus.

If a word or phrase has a high frequency TF in an article and rarely appears in other articles, it is considered that this word or phrase has a good ability to differentiate between categories and is suitable for classification.TF-IDF is actually: TF * IDF, TF Term Frequency, IDF Inverse Document Frequency (Inverse Document Frequency). If a phrase appears frequently in a class of documents, it means that the phrase can well represent the characteristics of the text of this class, and such phrases should be given a higher weight and selected as the feature words of the text of the class to distinguish it from other classes of documents.

Firstly, term frequency statistics are performed. Term frequency (TF) indicates how often a word (keyword) appears in the text. This number is usually normalized (usually word frequency divided by the total number of words in the text) to prevent it from biasing long documents.

$$\text{Term frequency}=\frac{\text{The number of times}\;a\;\text{word appears in an article}}{\text{Total number of words in the article}}$$

The second step is to calculate the IDF, which is the Inverse Document Frequency. The IDF of a particular word can be obtained by dividing the total number of documents by the number of documents containing the word, and then taking the logarithm of the quotient obtained. If the fewer documents containing a word, the larger the IDF, the better the ability to distinguish between categories. If a word is more common, then the denominator is larger, and the inverse document frequency is smaller and closer to 0. The reason for adding 1 to the denominator is to avoid the de-nominator being 0 (i.e., all documents do not contain the word). Log means the logarithm of the resulting value.

$$\mathrm{IDF}=\log\;\frac{\text{Total number of documents in the corpus}}{\text{Number of documents containing the word}+1}$$

Finally, we compute the TF-IDF.

TF−IDF=TF×IDF

It can be seen that TF-IDF is proportional to the number of occurrences of a word in a document and inversely proportional to the number of occurrences of the word in the whole corpus. From this, we can get the topic word for each example.

### 3.3 Input sequence construction

Segmentation processing: sentences and topic words are segmented using BERT’s own tokenizer. This step splits the text into subword units that are understood by the BERT model and adds special tokens [e.g., (CLS) and (SEP)].

Adding special tokens: the (CLS) token is added at the beginning of the sentence for the output representation of the classification task. Then, (SEP) markers are added at the end of the sentence question and answer, respectively and finally the topic word is added.

(CLS) question (SEP) sentence (SEP) topic word (SEP).

Positional encoding: BERT uses positional encoding to maintain information about the order of words in a sequence. After the addition of special tokens and topic words, the positional encoding is updated to ensure that the position of each word in the sequence is correctly represented.

### 3.4 Model training

Training process: during the training process, the model learns how to map the textual information in the input sequence (including the document body content and the subject words) to the output space of the target task. Since we have explicitly added topic words to the input sequence, the model is expected to better learn the association between this key information and the task goal. Fine tuning the BERT model using the transformed input sequences allows the model to learn specific relationships between the topic words and document content.

Hyper-parameter tuning: during the training process, we adjust hyper-parameters such as learning rate, batch size, and number of training rounds to obtain the best model performance.

## 4 Experiments

### 4.1 Datasets

In our experiments, we leverage the widely-used ACE 2005 dataset, which stands as a cornerstone in the field of information extraction and particularly in event extraction research. The Automatic Content Extraction (ACE) program, initiated by the Linguistic Data Consortium (LDC) and DARPA, aimed to facilitate the development of automatic systems capable of extracting meaningful information from unstructured text. The ACE 2005 dataset, in particular, has garnered significant attention due to its comprehensive annotation scheme and rich linguistic resources.

While more recent datasets, such as TAC-KBP, have emerged as valuable resources for event extraction, we chose ACE 2005 for several reasons. First, ACE 2005 provides a well-established benchmark with extensive annotations, enabling a direct comparison with prior work. Second, its fine-grained event subtypes and role classes align closely with our research objectives, particularly in exploring the impact of topic words on event extraction. TAC-KBP, while larger and more recent, focuses more on knowledge base population and entity linking, which are less central to our study. Additionally, ACE 2005’s BIO annotation schema (Ji and Grishman, 2008; Li et al., 2013; Yang and Mitchell, 2016; Nguyen et al., 2016; Chen et al., 2015) offers a precise method for marking event triggers and arguments, making it ideal for evaluating sequence labeling tasks.

The ACE 2005 dataset not only annotates 33 distinct event subtypes but also encompasses 36 role classes (Ji and Grishman, 2008; Li et al., 2013; Yang and Mitchell, 2016; Nguyen et al., 2016; Chen et al., 2015), which enable a fine-grained understanding of the events and their participants. These event subtypes cover a broad spectrum of real-world occurrences, ranging from physical events like “attack” and “die” to social and economic phenomena such as “elect” and “merge.” In Table 1, we provide a detailed breakdown of event types and their associated subtypes in ACE 2005.

**Table 1:** Event types and subtypes in ACE 2005.

Event type	Subtypes
Justice	Arrest, Charge, Trial, Sentence, Appeal
Attack	Bombing, Shooting, Assault, Raid
Business	Merge, Start-Org, End-Org, Declare-Bankruptcy
Conflict	Protest, Riot, War, Demonstrate
Life	Be-Born, Marry, Divorce, Die
Movement	Transport, Transfer-Ownership
Contact	Phone-Write, Meet

The diversity of events represented in the dataset underscores its importance for evaluating the robustness and generalizability of event extraction systems. Furthermore, the ACE 2005 dataset employs the BIO annotation schema (Ji and Grishman, 2008; Li et al., 2013; Yang and Mitchell, 2016; Nguyen et al., 2016; Chen et al., 2015), which is a widely adopted scheme for sequence labeling tasks. This scheme provides a precise method for marking the boundaries of event triggers and arguments within a text, allowing researchers to accurately evaluate the performance of their models.

The importance of the ACE 2005 dataset lies not only in its extensive annotation but also in its role as a benchmark for evaluating the progress in event extraction research. Over the years, numerous studies have utilized this dataset to develop and evaluate their methods, fostering a vibrant community of researchers working toward advancing the state-of-the-art in this field.

In our work, by conducting experiments on the ACE 2005 dataset, we are able to position our proposed method within the broader context of event extraction research and evaluate its effectiveness against well-established baselines. The results obtained on this dataset provide compelling evidence of the effectiveness of our approach in leveraging topic words to enhance event extraction performance. Future work will explore the applicability of our method to larger and more diverse datasets, such as TAC-KBP, to further validate its scalability and generalizability.

### 4.2 Baseline models

We conducted a comprehensive performance evaluation of our framework against several prominent preceding models. dbRNN (Su and Kuo, 2018), a framework rooted in LSTM technology, leverages dependency graph information to meticulously extract event triggers and parameter roles. Joint3EE, on the other hand, embodies a multi-tasking prowess, adeptly handling entity recognition, trigger detection, and role assignment through a shared bidirectional GRU (BI-GRU) hidden representation layer. GAIL-ELMo, an innovative fusion of Generative Adversarial Imitation Learning (GAIL) and Embeddings from Language Models (ELMo), aims to bolster GAIL’s performance in text processing endeavors by harnessing ELMo’s robust semantic representation capabilities (Zhang et al., 2019). Alternatively, the imitation learning mechanism inherent in GAIL can be fruitfully applied to enhance the training of ELMo-based models. Dy-GIE++, an advanced model for entity, relationship and event extraction, relies on context span representation to effectively capture intricate contextual nuances surrounding entities, relationships, and events within textual data (Wadden et al., 2019). Leveraging the Al-lenNLP framework, it excels at deciphering complex contextual information.

As Table 2 meticulously illustrates, our model, designated as BERT_QA_Trigger, demonstrates a superior trigger detection strategy, outperforming the baseline system by a notable margin. This underscores the efficacy and robustness of our approach in identifying and extracting event triggers with precision.

**Table 2:** Trigger detection results.

Trigger detection scenarios	**Trigger identification**			**Trigger ID + classification**		
	P	R	F1	P	R	F1
BEAR_QA_Arg	53.48	34.72	42.11	35.65	17.36	23.35
BEAR_QA_Arg (with topic words)	80.86	34.72	48.59	53.91	17.36	26.67
w/o dynamic threshold	87.71	34.72	49.75	43.85	17.36	24.88
w/o dynamic threshold (with topic words)	49.83	52.08	50.93	33.22	34.72	33.95

### 4.3 Evaluation metrics

The scoring metric is the F1 measure. The F1 measure is defined as F1 = 2 × P × R/(P + R), where P is the precision, and R is the recall. Precision measures the fraction of automatically discovered relations which were correct over all the identified relations. Recall measures the fraction of relations that were identified over all relations that exists and should be identified in the text (Rink et al., 2011).

F1 metric: a comprehensive evaluation metric.

This formula combines two indicators, precision rate and recall rate.

An F1 metric, also known as an F1 score or F1 score, is a widely used evaluation metric that combines accuracy and recall into a single score to evaluate a model’s performance (Rainio et al., 2024). It is an important evaluation index in natural language processing tasks, especially in event extraction tasks. It is particularly suitable for tasks where accuracy and recall are both important considerations. In the context of event extraction, the F1 metric provides an overall assessment of the model’s recognition and extraction of relevant event information from unstructured text. The F1 measure is defined as the harmonic average of precision and recall, where:

$F1=\frac{2\times P\times R}{P+R}$ a balance point, so that the model can accurately identify the target relationship, but also cover all the relationships that should be identified as much as possible. F1 scores are in the range [0, 1], where 1 represents perfect performance (i.e., 100% accuracy and recall) and 0 represents worst performance (i.e., at least one of the accuracy and recall rates is 0).

Precision (P): accuracy measures the accuracy of the model’s predictions. Specifically, it is the automatic discovery of the correct part of the relationship among all the relationships identified by the model (Rink et al., 2011). In other words, it measures how many of the events extracted by the model are actually relevant.

$$\text{Precision}=\frac{\mathrm{TP}}{\mathrm{TP}+\mathrm{FP}}$$

A high accuracy rate means that there are fewer cases in which the model incorrectly labels non-relationships as relationships when identifying relationships, but it can also cause the model to be too conservative and fail to identify all the relationships that actually exist.

Recall rate (R): recall rate measures the completeness of model predictions. It is the part of the relationship that the model correctly identifies among all the relationships that actually exist and should be identified in the text (Rink et al., 2011). In the context of event extraction, the recall rate represents the number of actual events in the text successfully extracted by the model.

$$\text{Recall}=\frac{\mathrm{TP}}{\mathrm{TP}+\mathrm{FN}}$$

The high recall rate means that the model can recognize most of the actual relationships in the text, but it may also cause the model to misidentify some non-relationships as relationships, that is, sacrifice a certain accuracy rate.

The importance of event extraction.

Event extraction aims to identify and extract structured information about events from unstructured text, such as news articles or social media posts. The complexity and diversity of event expression, as well as the ambiguities and contextual dependencies of language, pose significant challenges to this task (Liu and Luo, 2024). Therefore, evaluating the performance of event extraction models is critical to understanding their advantages and limitations and to improving their effectiveness.

F1 metrics provide a balanced assessment of accuracy and recall, both of which are fundamental considerations in event extraction (Kuhn and Johnson, 2013). A high precision score indicates that the model is making accurate predictions, while a high recall score indicates that the model is recognizing most relevant events in the text. By combining these two metrics into a single F1 score, a more comprehensive and balanced assessment perspective can be provided.

### 4.4 Experiment setup

For the hyperparameters, we chose a learning rate of 4 × 10^−5^, which allows the model to gradually adjust the parameters in smaller steps, resulting in a more stable training process and better convergence. When the batch size is set to 60, the model benefits from the noise while maintaining a relatively stable training process. With a maximum sequence length of 180, the model is able to capture meaningful long term dependencies while remaining efficient.

### 4.5 Results

Evaluation on ACE Event Extraction We compare the performance of our model with the previous one (Du and Cardie, 2020).

As shown in Tables 3, 4, we give a comparison of the performance of our model with that of the model without added topic words.

**Table 3:** Trigger detection results.

Trigger detection scenarios	Trigger identification			Trigger ID + classification		
	P	R	F1	P	R	F1
DbRNN	–	–	–	71.20	66.80	68.90
Joint3EE	68.30	72.10	70.10	65.50	69.20	67.30
GAIL-ELMo	74.20	71.20	73.90	74.80	69.40	72.00
DYGIE++, BERT + LSTM	73.60	68.40	70.90	72.10	67.30	69.60
BEAR_QA_Trigger (with topic words)	75.40	74.22	74.80	77.88	76.67	77.27

**Table 4:** Performance comparison of trigger detection with and without topic words.

Trigger detection scenarios	Trigger identification			Trigger ID + classification		
	P	R	F1	P	R	F1
BEAR_QA_Trigger	74.29	77.42	75.82	71.12	73.70	72.39
BEAR_QA_Trigger (with topic words)	75.40	74.22	74.80	77.88	76.67	77.27

Based on Tables 3, 4, we can evaluate and analyse the performance results of the experiments as follows:

In the trigger recognition task, we observe a significant performance difference, which is reflected in the change of F1 scores when using different trigger words. When using only BERT_QA_Trigger, the F1 score of the model is 72.39%. However, when topic words are added, i.e., BERT_QA_Trigger (with topic words), the F1 score improves to 77.27%, which indicates that the addition of topic words helps the model to identify trigger words more accurately and improves the recognition performance.

In the extracting arguments task, the results are more complex. Without dynamic thresholding, the model’s F1 score is relatively low, and it especially performs poorly in terms of argument extraction accuracy, which increases significantly with the addition of topic words, with the F1 value growing from 42.11 to 48.59%. When dynamic thresholding is introduced, the model shows a significant improvement in F1 scores, especially after the addition of topic words the performance improves significantly.

The addition of topic words positively affects the model performance in both trigger recognition and argument extraction tasks, which suggests that topic words have an important role as contextual information in NLP tasks, and can help the model better understand the text content and improve the task performance.

Meanwhile, to further demonstrate the applicability of our method in the professional field, we propose a case study from the field of physics journals. Table 5 illustrates how our method effectively extracts key information from scientific texts, demonstrating its generality and robustness in handling domain specific languages and complex event structures. The use of keywords such as “particles” and “quantum computing” guides the model to focus on the most relevant aspects of the event, improving accuracy and recall.

**Table 5:** Physics journal example.

Event type	Example
Input text	“The research team discovered a new particle, which could revolutionize quantum computing.”
Extracted topic words	“particle,” “quantum computing”
Question Template	“What did the research team discover?”
Input text	“The terrorist group launched a coordinated attack on several government buildings.”

In summary, the model in this experiment shows better performance in both trigger recognition and argument extraction tasks. Our model achieves significant improvements over the baseline model without topic words. The model in this paper achieves relatively good results on the dataset ACE 2005.

In this paper, we tackle the challenging task of event extraction, which aims to identify and extract structured information about events from unstructured text. Despite significant progress, event extraction remains difficult due to the complexity and diversity of event expressions, as well as the inherent ambiguity and context-dependency of language. To address these challenges, we propose a novel approach that incorporates topic words and leverages the power of answering almost natural questions. By framing event extraction as a question answering task and constructing informative questions tailored to specific event types and their arguments, we guide the model to focus on relevant information and filter out noise. Our results demonstrate that this approach effectively enhances the precision and recall of event extraction, suggesting that leveraging topic words and question answering techniques can pave the way for more accurate and robust event extraction systems.

## 5 Analysis

While our proposed approach of incorporating topic words into the input sequence for event extraction demonstrates promising results, several challenges and limitations remain. These issues highlight areas for future improvement and provide valuable insights into the model’s behavior under different conditions. Below, we analyze the key errors observed during our experiments, discuss their implications, and propose potential solutions.

### 5.1 Topic word selection errors

The accuracy of topic word extraction is critical to the model’s performance. However, we identified two primary issues in this process:

Inaccuracy: in some cases, the extracted topic words were not sufficiently representative of the event context. For example, in the financial news domain, the topic word “market” was frequently extracted alongside “earnings” in the sentence, *“The company announced its quarterly earnings, which exceeded market expectations.”* While “earnings” is directly related to the event, “market” is a more general term that introduces noise, potentially misleading the model and reducing extraction precision.

Ambiguity: certain words have multiple meanings depending on the context, making it challenging for the model to disambiguate their correct interpretation. For instance, the word “bank” could refer to a financial institution or the side of a river, depending on the surrounding text. This ambiguity can lead to incorrect event associations, particularly in domains with polysemous terms.

Proposed solution: to address these issues, we recommend integrating domain-specific ontologies or knowledge graphs into the topic word extraction process. These resources can provide additional contextual cues, helping the model distinguish between relevant and irrelevant terms. Additionally, leveraging pre-trained language models fine-tuned on domain-specific corpora may improve the accuracy of topic word selection.

### 5.2 Question template limitations

The question templates used to guide event extraction also present challenges:

Rigidness: the current templates may lack the flexibility needed to capture the nuances of different event types and their arguments. For example, a rigid template like *“Who attacked whom?”* may fail to capture additional event parameters such as location or time, leading to incomplete extraction.

Incompleteness: in some cases, the templates do not cover all necessary aspects of an event. For instance, in the sentence *“The terrorist group launched a coordinated attack on several government buildings,”* the template failed to capture the location of the attack, resulting in an incomplete event extraction.

Proposed solution: future work should focus on developing dynamic question templates that adapt to the specific requirements of each event type. Incorporating multi-turn question-answering frameworks could also enable the model to iteratively refine its understanding of complex events.

### 5.3 Model limitations

Despite the use of advanced pre-trained language models like BERT, several model-specific limitations were observed:

Overfitting: when trained on small or specialized datasets, the model exhibited signs of overfitting, achieving high F1 scores on the training set but performing poorly on unseen data. This issue is particularly pronounced in domains with limited annotated data, such as niche scientific fields.

Contextual understanding: while pre-trained models excel at capturing local contextual information, they often struggle with long-range dependencies and cross-sentence event coreference. For example, in document-level event extraction, the model may fail to associate related events mentioned in different paragraphs.

Scalability: the computational cost of processing large-scale datasets with thousands of event types remains a significant challenge. As the volume of data increases, the model’s performance may degrade due to resource constraints.

Proposed solution: to mitigate overfitting, we suggest employing data augmentation techniques or transfer learning from larger, more diverse datasets. For long-range dependencies, document-level context modeling techniques, such as hierarchical attention mechanisms, could be explored. Additionally, optimizing the model’s architecture for scalability, such as through distributed training, may help address computational limitations.

### 5.4 Language and domain specificity

The model’s performance is currently evaluated primarily on English texts, and its applicability to other languages or domains remains untested. Linguistic differences and variations in event expressions across languages may pose additional challenges.

Proposed solution: expanding the model’s training to include multi-lingual corpora and evaluating its performance in diverse linguistic contexts could enhance its generalizability. Techniques such as cross-lingual transfer learning may also prove beneficial.

### 5.5 Case studies

To better illustrate the errors and limitations of the proposed approach, we present the following case studies (Table 6):

**Table 6:** Inaccurate topic word extraction.

Event type	Example
Input text	“The company announced its quarterly earnings, which exceeded market expectations.”
Extracted topic words	“earnings,” “market”
Question template	“What did the company announce?”
Answer	“The company announced its quarterly earnings.”

Table 6 shows case study 1: inaccurate topic word extraction, when the input text is “The company announced its quarterly earnings, which exceeded market expectations.” The extracted subject words are “earnings” and “market.” The error that arises from this example is that the topic word “market” is not directly related to the event “company announced earnings.” It is a general term that appears frequently in financial news. The inclusion of “market” as a topic word may introduce noise and reduce the precision of event extraction.

Table 7 shows case study 2: incomplete question template. The error in this case is that the question template does not capture the location of the attack, which is an important parameter for the “Attack” event type. This resulted in an incomplete event extraction.

**Table 7:** Incomplete question template.

Event type	“Attack”
Question template	“Who attacked whom?”
Input text	“The terrorist group launched a coordinated attack on several government buildings.”

Case Study 3 is overfitting. When using a small specialized dataset and containing only a few event types and parameters, the model achieves high F1 scores on the training set but performs poorly on a held-out test set. This is because the model has overfit the training data and is unable to generalize to new, unseen event types and contexts.

## 6 Conclusion

In this study, we have presented a novel approach to enhancing event extraction by incorporating topic words into the input layer of an event extraction model. Our method leverages the contextual relevance of topic words to guide the model toward identifying events more accurately and comprehensively. The experimental results show that using topic words as additional input features significantly improves the performance of the event extraction model. The significant improvement in precision, recall, and F1 scores we observed highlights the effectiveness of our proposed approach. We envision that our approach can be extended and adapted to address other related tasks, such as entity linking and relation extraction, further advancing the state of art in natural language understanding.

## Data Availability

The original contributions presented in the study are included in the article/supplementary material, further inquiries can be directed to the corresponding author.
